# Comparative Analysis of Bovine Fecal Microbiota and Short-Chain Fatty Acids Variation During Dry Period, Pregnancy and Lactation

**DOI:** 10.3390/microorganisms14061268

**Published:** 2026-06-04

**Authors:** Morgan Obinna Okpara, Eleni Nikouli, Eleni Mente, Leonard Chidi Ugwuowo, Konstantinos Kormas

**Affiliations:** 1 Department of Ichthyology and Aquatic Environment, School of Agricultural Sciences, University of Thessaly, 384 46 Volos, Greece; morganokpara@gmail.com (M.O.O.); elnikoul@uth.gr (E.N.); 2 Department of Animal Science, Faculty of Agriculture, Nnamdi Azikiwe University, Awka 420110, Nigeria; lc.ugwuowo@unizik.edu.ng; 3 School of Veterinary Medicine, Aristotle University of Thessaloniki, 541 24 Thessaloniki, Greece; emente@vet.auth.gr

**Keywords:** Bunaji White Fulani cattle, rumen, gut microbiota, reproductive stages, host-microbe interactions

## Abstract

The bovine gut microbiota is crucial for many physiological functions, but how microbial interactions and related metabolic processes shift during pregnancy and lactation remains poorly understood. This study utilized fecal samples from 18 Bunaji (White Fulani) cows as a model to examine the gut microbial composition and structure across lactation, pregnancy, and the dry phase using 16S rRNA gene sequencing. Community composition analysis, alpha and beta diversity, LEfSe and correlation analyses were performed to explore the relationship between these reproductive stages, gut microbiota, and concentrations of short-chain fatty acids (SCFAs). Based on the Shannon and Simpson alpha diversity indices, no significant differences among the groups were found. However, beta diversity analysis revealed clear distinctions in microbial community structures between the groups. The most abundant microbial phyla across all three groups were the Bacillota (55–60%) and Bacteroidota (25–33%). At both the family and genus levels, cellulose-degrading bacteria such as Oscillospiraceae, Bacteroidaceae, Sphingobacteriaceae, *Intestinimonas*, *Bacteroides*, and *Acetivibrio* were prevalent across lactating, pregnant, and dry cows. Fifteen genera, including *Intestinimonas*, *Bacteroides*, *Aristaeella*, and *Acinetobacter*, were identified as potential biomarkers due to their significantly different abundances (*p* < 0.05) among the groups based on LEfSe. Notably, Spearman’s correlation analysis (*p* < 0.05) showed significant associations between the levels of specific microbial taxa and SCFA concentrations. In conclusion, although the core gut microbiota was similar across the groups, significant variations in microbial composition were detected. Additionally, these microbial profiles were associated with variations in fecal SCFA levels, indicating a potential interaction between them.

## 1. Introduction

The gastrointestinal tract (GIT) in cattle plays a central role in digesting, absorbing, and secreting nutrients, and it hosts trillions of microorganisms. These microbial populations are heavily shaped by factors such as diet, age, breed, genetics, and geography [1,2,3]. In forage-based diets, much of the fiber is fermented and degraded in the rumen, while other nutrients are absorbed in various sections of the GIT. Gut microbes are vital for converting fiber into compounds that the host can absorb. This microbial activity influences not only fetal development and maternal metabolic stability but also the metabolism of bile acids, hormones, inflammatory agents, and short-chain fatty acids (SCFAs), which are an important energy source [4,5,6]. Emerging research emphasizes the role of gut bacteria in regulating physiological functions and metabolic shifts during pregnancy in both humans and animals [7,8,9,10].

Pregnancy and lactation bring about complex hormonal, immune, and metabolic shifts that support fetal growth and offspring health [11,12]. Human studies have shown that gut microbiota composition can change during pregnancy, potentially affecting immune responses and metabolism [13,14,15]. In pigs, the gut microbiome significantly affects milk composition during lactation and is closely tied to maternal metabolism throughout both pregnancy and lactation [3]. However, some studies suggest that the gut microbiota remains stable during pregnancy [16,17,18], indicating that more research is needed to clarify microbial and metabolic dynamics during these phases.

Cattle serve as an excellent model for studying gut microbiota and host interactions because of their specialized digestive system. The rumen, in particular, acts as a fermentation chamber containing bacteria, archaea, fungi, and protozoa that help break down plant materials and produce volatile fatty acids (VFAs), which are essential for energy production and overall health [19,20,21]. Investigating gut microbial changes and SCFA production during pregnancy and lactation in cows can reveal how the microbiome impacts not only digestion and immunity but also host physiology more broadly. The present study focused on Bunaji cows, a West African indigenous breed known for high milk production, rapid growth, and adaptability to diverse forage sources. The research aimed to analyze gut microbial shifts and SCFA levels during pregnancy, lactation, and the dry (neither pregnant nor lactating) period. By comparing SCFA concentrations across these physiological states, we sought to understand how gut microbial composition varies with reproductive stage.

Although previous studies have examined the relationship between cows’ gut microbiota and factors such as diet, metabolism, and environment [19,22], limited data exist on how these microbial communities and their metabolites shift across the reproductive cycle. To fill this gap, we investigated the fecal microbiota and SCFA profiles in Bunaji cows, hypothesizing that both pregnancy and lactation influence gut microbial composition and SCFA production. Fecal microbiota is a valid and practical proxy for the gut, but not the rumen, habitat of cattle [23]. In this study, the fecal microbiota was used as the only available non-invasive practice for similar analyses.

## 2. Materials and Methods

### 2.1. Experimental Site, Management of Animals and Sample Collection

This study involved 18 healthy Bunaji cows in their second parity, randomly chosen from a herd of approximately 500 animals on a farm located in Obinze, Owerri, Nigeria (coordinates: 5°24′59.99″ N; 6°57′59.99″ E). Animals were raised in a free-range production system. They grazed year-round on natural shrub grassland pastures and had unlimited access to forage and clean drinking water. The pasture was mostly made up of herbage species, like *Panicum maximum*, *Pennisetum purpureum*, *Cynodon dactylon*, *Digitaria decumbens*, *Brachiaria decumbens*, *Calopogonium mucunoides*, *Centrosema pubescens* and *Stylosanthes guianensis*, and shrubs like *Gliricidia sepium*, *Leucaena leucocephala*, *Chromolaena odorata* and *Aspilia africana*. This seasonal plant composition occurred for several weeks prior to sample collection. Cows that had been administered antibiotics prior to our sampling or had shown signs of fever, weakness, or abnormal behavior within one week before sampling were excluded from the study. The cows were categorized into three physiological groups: pregnant, lactating, and dry (neither pregnant nor lactating), with six cows per group. The pregnant cows had similar breeding dates and were in their late stage of pregnancy, while the lactating cows were in their late stage of lactation. Fresh fecal samples (~200 g per animal) were collected from each cow on a single sampling day using the rectal grab method or during spontaneous release. For rectal grabs, sterile disposable gloves were worn, and a gloved hand was inserted into the rectum to retrieve feces directly. A new pair of gloves was used for each animal to avoid cross-contamination. Each sample was thoroughly mixed and split into two portions: one stored in a sterile 2 mL centrifuge tube for genomic DNA extraction, and the other in a sterile 50 mL tube for short-chain fatty acid (SCFA) analysis. Samples were kept on ice during transport to Inqaba Biotech West Africa Laboratory in Ibadan, Nigeria, for processing.

### 2.2. Chemical Composition of Forages

Representative fresh forage samples were randomly harvested from different points within the pasture to obtain a composite sample reflecting the botanical composition of the grazing area. The samples were cleaned to remove debris, chopped into smaller pieces, and oven-dried at 65 °C for 48–72 h to constant weight. The dried samples were then ground to pass through a 1 mm sieve and stored in airtight containers for laboratory analysis. The (AOAC, 930.15) method was used to measure dry matter (DM) at 135° for 3 h. Kjeldahl digestion (AOAC, 984.13), distillation, and titration methods were used to measure nitrogen. To determine the crude protein (CP) value, the nitrogen content was multiplied by 6.25 (CP = N × 6.25). The detergent fiber analysis method described by Van Soest et al. [24] was used to determine neutral detergent fiber (NDF) and acid detergent fiber (ADF). The determination of ash content was done in accordance with AOAC, 942.05 [25].

### 2.3. DNA Extraction and Sequencing

Genomic DNA extraction and subsequent library preparation were performed concomitantly from six fecal samples per group using the Quick-DNA™ Fecal/Soil Microbe Miniprep Kit (ZymoResearch, Irvine, CA, USA), following the manufacturer’s instructions. The number of replicate samples per treatment was set as the maximum we could sample from, having as many individuals as possible with similar dietary habits and developmental stage, although a higher number of replicates could further eliminate the individual variability of the animals’ gut microbiota. The bacterial community composition was assessed via 16S rRNA gene amplicon sequencing, focusing on the V3–V4 region, amplified using primers S-D-Bact-0341-b-S-17 and S-D-Bact-0785-a-A-21 [26]. PCR amplification and sequencing were conducted at MR DNA (Shallowater, TX, USA) using an Illumina MiSeq platform with paired-end reads (2 × 300 bp), following standard protocols.

### 2.4. Bioinformatics and Data Analysis

The 16S rRNA sequencing raw data were processed using the MOTHUR MiSeq SOP procedure [27]. Raw sequence reads have been deposited in the Short Read Archive (https://www.ncbi.nlm.nih.gov/sra; BioProject access number PRJNA1439808). The operational taxonomic units (OTUs) at a 97% cutoff similarity level were classified with the SILVA database release 138.2 [28]. Alpha diversity metrics, including Chao1, observed OTUs, Shannon, and Simpson indices, were used to assess within-sample diversity. Beta diversity was determined using PERMANOVA (Permutational Multivariate Analysis of Variance) based on Bray–Curtis and Jaccard distance metrics. To identify significantly different bacterial taxa between groups, the LEfSe (Linear Discriminant Analysis Effect Size) method [29] was applied using an LDA threshold >2 and *p*-value < 0.05. Statistical and microbiome data analyses, including both qualitative and quantitative assessments, were performed using the MicrobiomeAnalyst web platform [30]. Visual representations of microbial taxa at the phylum, family, and genus levels were created using ChiPlot (https://www.chiplot.online/, accessed on 2 April 2025). The Shapiro–Wilk test was used to assess data normality. Group differences in microbial relative abundance and SCFA concentrations were analyzed via one-way ANOVA in R (v. 4.4.2). Correlations between SCFA levels and predominant microbial genera were assessed using Spearman’s rank correlation, also in R (v. 4.4.2). Statistical significance was set at *p* < 0.05, *p* < 0.01, and *p* < 0.001.

### 2.5. SCFA Analysis

To quantify SCFAs, 0.1 g of each fecal sample was mixed thoroughly in 25% phosphoric acid (4:1, *v*/*v*) in a 5 mL tube. Concentrations of various SCFAs—including acetate, propionate, butyrate, isobutyrate, isovalerate, valerate, and caproate—were measured using gas chromatography based on the method described by Guan et al. [31]. SCFA levels were expressed in micromoles per gram (μmol/g) of fresh fecal matter.

## 3. Results

The chemical composition of forages from the pasture used in this study had a high percentage of carbohydrates, with most of this fraction being complex, fermentable sugars (Table 1).

### 3.1. Gut Bacterial Microbiota

After quality filtering of the 16S rRNA gene (V3–V4 region) amplicon sequencing data, a total of 1,746,455 reads were retained. Per-sample read counts ranged between 80,749 and 115,042. Reads were assigned to 341 OTUs in total. The five most abundant phyla were Bacillota, Bacteroidota, Verrucomicrobiota, Spirochaetota, and Pseudomonadota. Dominant families included Oscillospiraceae, Bacteroidaceae, Sphingobacteriaceae, Rikenellaceae, and Lachnospiraceae, while the most prevalent genera were *Intestinimonas*, *Bacteroides*, *Acetivibrio*, *Lysinibacillus*, and *Akkermansia*.

**Figure 1:**
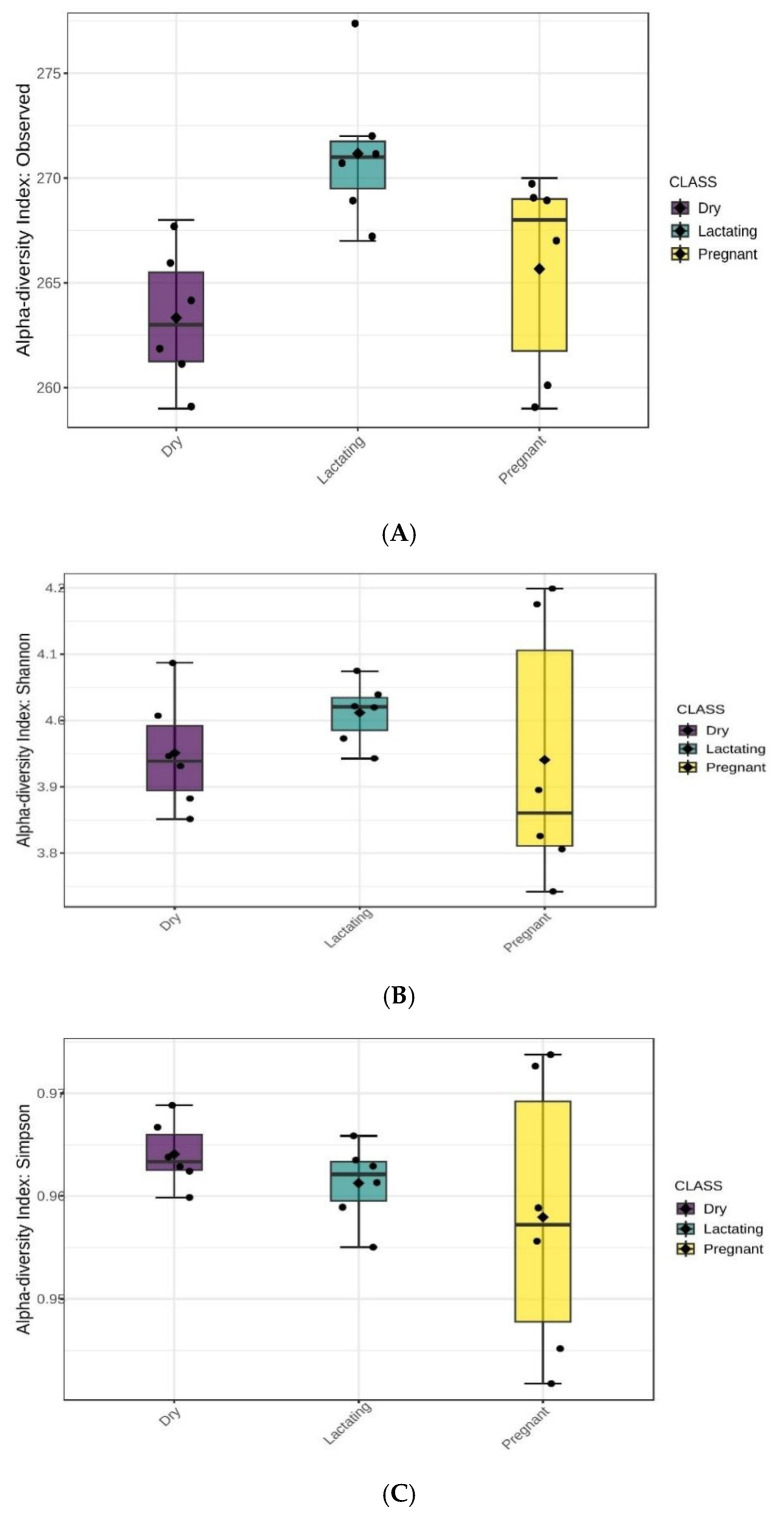
Alpha diversity boxplot of gut microbial community based on (**A**) Observed; (**B**) Shannon index; (**C**) Simpson index. (*p* = 0.014 for Observed, *p* = 0.36 for Shannon index and *p* = 0.44 for Simpson index).

Observed species, Shannon, Simpson, and Chao1 were used to assess microbial diversity across pregnant, lactating, and dry cows (Table 2; Figure 1A–C). Observed species counts showed statistically significant differences (*p* ≤ 0.05), being lowest in dry cows and highest in lactating ones. The Shannon index showed slight, non-significant differences (*p* ≥ 0.05), with pregnant cows having marginally higher values. Simpson index trends were similar to Shannon; pregnant cows had the highest values, implying lower evenness, while lactating cows had the lowest, indicating more even distribution. The Chao1 index, used to estimate species richness, was highest in lactating cows but not statistically different across groups (*p* > 0.5).

**Figure 2:**
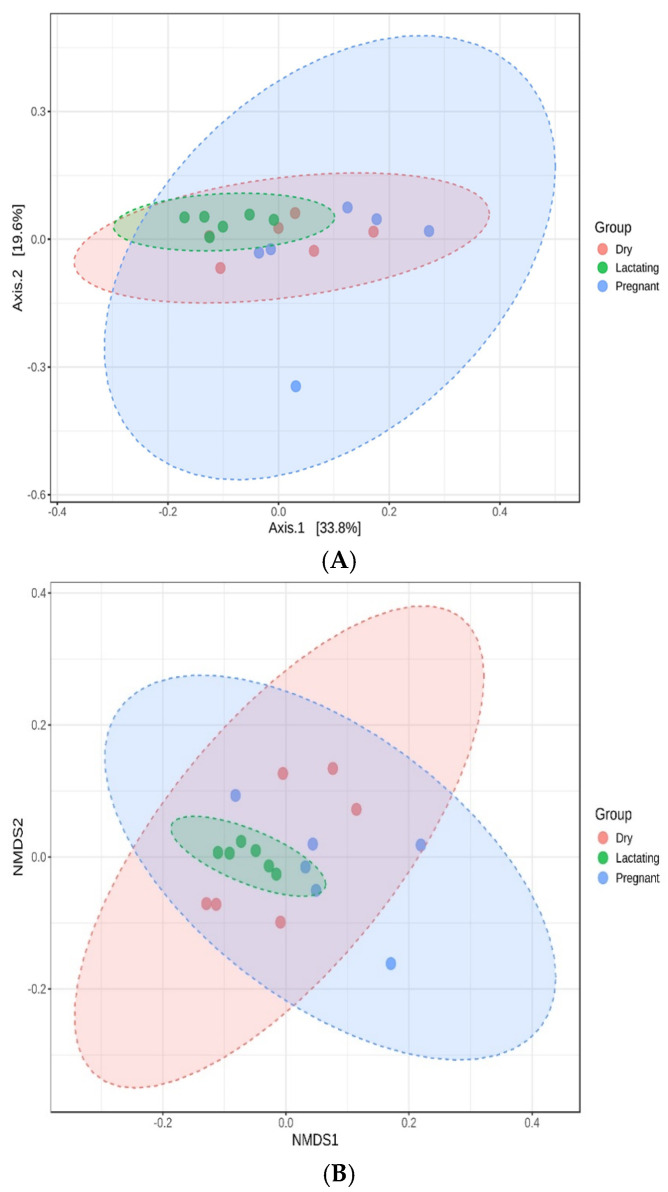
Principal coordinates analysis (PCoA) by (**A**) Bray-Curtis distance and with PERMANOVA statistics (R^2^ = 0.262; *p* = 0.002) and (**B**) Non-metric Multidimensional Scaling (NMDS) based on the Jaccard distance and PERMANOVA statistics (R^2^ = 0.221; *p* = 0.001) between gut microbiota of dry, lactating and pregnant cows.

Inter-group differences were assessed using beta diversity metrics via Principal Coordinates Analysis (PCoA with Bray–Curtis distance) and Non-Metric Multidimensional Scaling (NMDS with Jaccard distance) (Figure 2A,B). The three physiological groups showed overlapping clustering patterns. However, based on PERMANOVA, statistically significant differences between the groups but with low R^2^ values were found, suggesting distinct gut microbial community structures.

**Figure 3:**
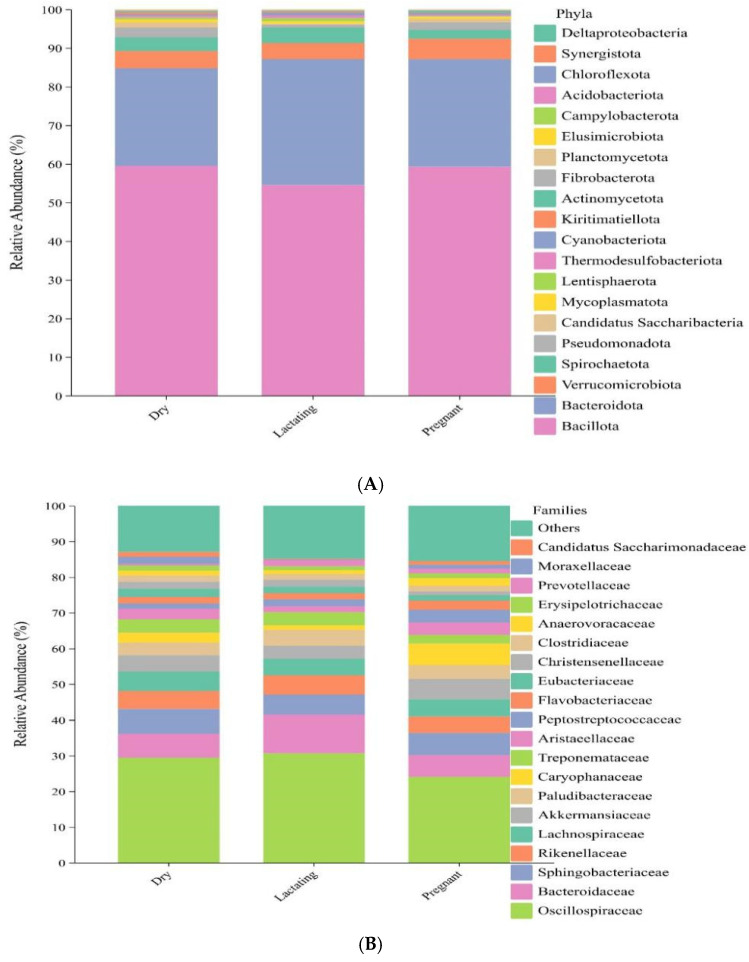

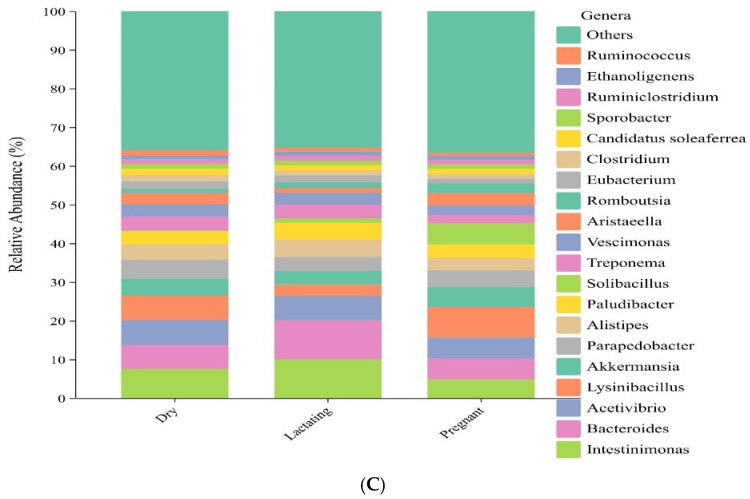
A comparison of the gut microbiota of cows at several taxonomic levels. The relative abundance illustrates dominant phyla (**A**), families (**B**) and genera (**C**).

Across all samples, 20 phyla, 133 families, and 341 genera were detected. Bacillota (55–60%) and Bacteroidota (25–33%) dominated all groups, accounting for 85–87% of reads (Figure 3A–C). Other dominant phyla included Verrucomicrobiota, Spirochaetota, Pseudomonadota, Candidatus Saccharibacteria, Mycoplasmatota, Lentisphaerota, Thermodesulfobacteriota, and Cyanobacteriota. Bacteroidota was significantly more abundant in lactating cows and least abundant in dry cows (*p* < 0.05). Pseudomonadota abundance was significantly higher in dry cows than in other groups (*p* < 0.05), while the other dominant phyla showed no significant differences (*p* > 0.05). Similarly, Lentisphaerota showed the highest abundance in lactating cows and the lowest in pregnant cows (*p* < 0.05) (Table 3).

At the family level, 133 families were identified. Oscillospiraceae (24–31% in the whole dataset), Bacteroidaceae (6–11%), and Sphingobacteriaceae (6–7%) were predominant (Figure 2B). Additional prevalent families included Rikenellaceae, Lachnospiraceae, Akkermansiaceae, Paludibacteraceae, Caryophanaceae, Treponemataceae, and Aristaeellaceae. Oscillospiraceae and Bacteroidaceae were most abundant in lactating cows and lowest in pregnant cows (*p* < 0.05). Aristaeellaceae were significantly more abundant in pregnant cows (*p* < 0.05). No significant differences were observed for other major families (*p* > 0.05) (Table 4).

Genus-level analysis revealed *Intestinimonas* (5–10%), *Bacteroides* (5–10%), *Acetivibrio* (6–7%), and *Lysinibacillus* (3–8%) as dominant genera (Figure 3C). Other notable genera included *Akkermansia*, *Parapedobacter*, *Alistipes*, *Paludibacter*, *Treponema*, *Vescimonas*, and *Aristaeella*. Clear differences were observed among physiological stages: *Intestinimonas* and *Bacteroides* were significantly higher in lactating cows (*p* < 0.05). No significant variation was found in the other listed genera (*p* > 0.05) (Table 5).

**Figure 4:**
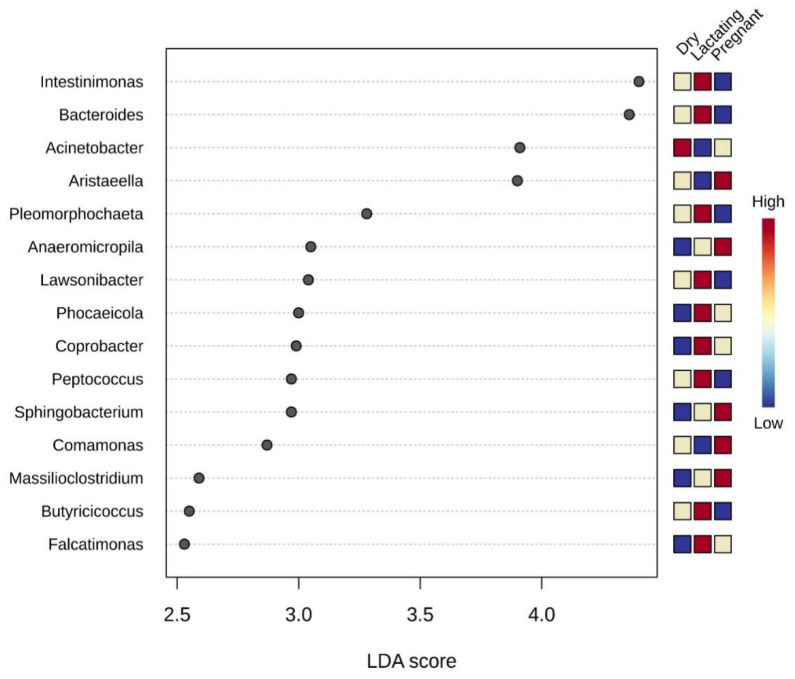
Statistically significant differences in relative abundance (LDA ≥ 2.5) between dry, lactating and dairy cows via LEfSe analysis at the genus level.

LEfSe analysis (LDA ≥ 2.5, *p* ≤ 0.05) identified taxa that significantly characterized each group. The dry group was marked by the genus *Acinetobacter*. The lactating group had multiple biomarkers, including *Intestinimonas*, *Bacteroides*, *Pleomorphochaeta*, *Lawsonibacter*, *Phocaeicola*, *Coprobacter*, *Peptococcus*, *Butyricicoccus*, and *Falcatimonas*. In pregnant cows, biomarkers included *Aristaeella*, *Anaeromicrophila*, *Sphingobacterium*, *Comamonas*, and *Massilioclostridium* (Figure 4).

### 3.2. SCFAs

**Figure 5:**
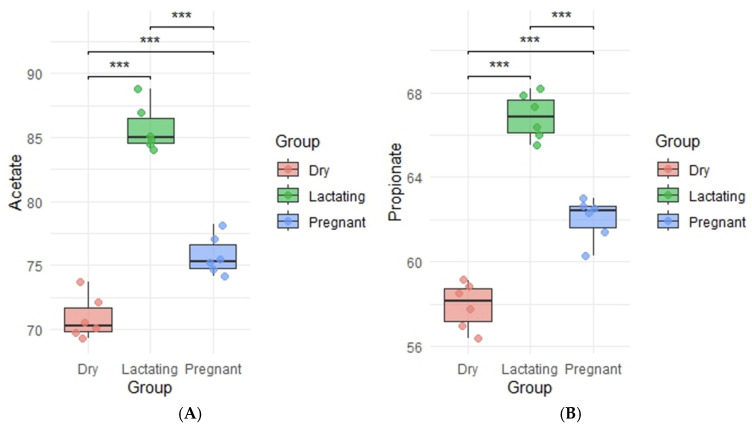

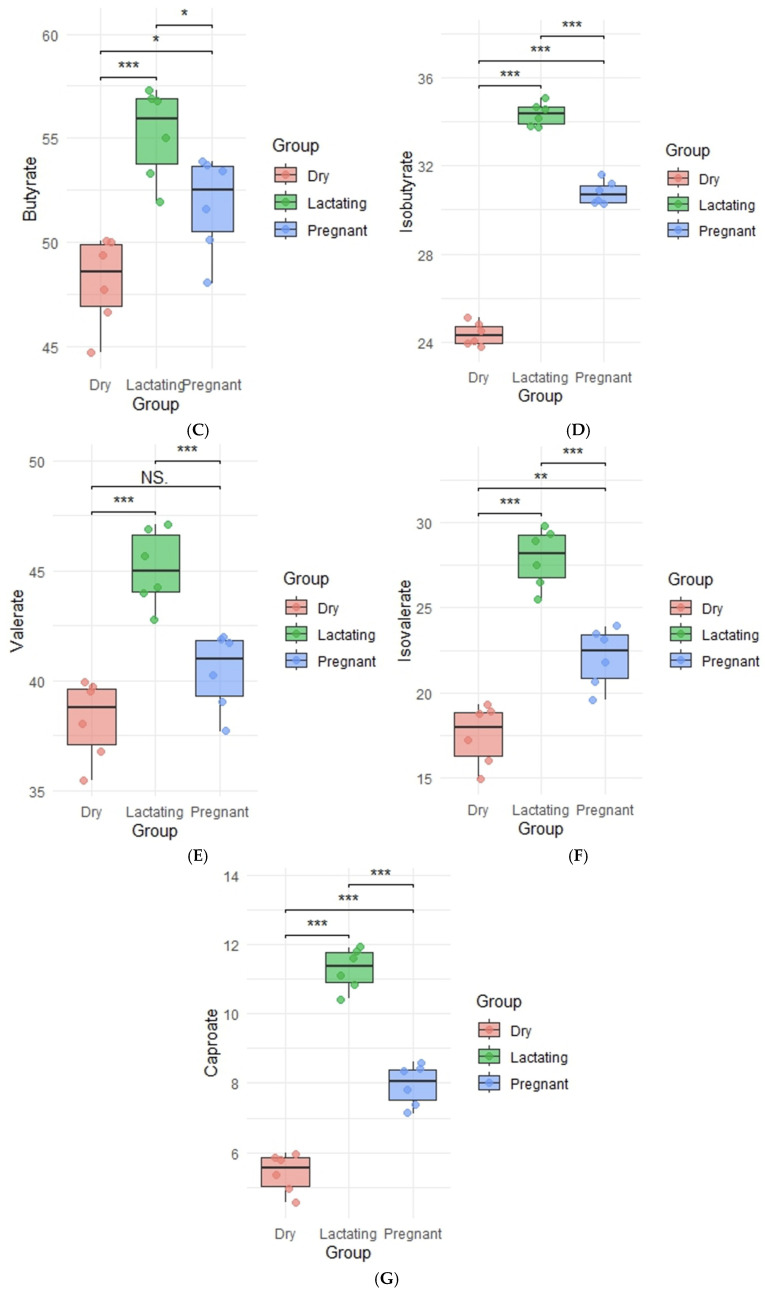
Boxplots show concentration of SCFA metabolites. The results of the target quantification of Acetate (**A**), Propionate (**B**), Butyrate (**C**), Isobutyrate (**D**), Valerate (**E**), Isovalerate (**F**), and Caproate (**G**). * *p* < 0.05, ** *p* < 0.01, *** *p* < 0.001. NS: not significant.

The SCFAs composition in cows from the three groups is shown in Figure 5A–G. Concentrations of acetate, propionate, butyrate, isobutyrate, valerate, isovalerate and caproate were determined, and univariate analysis demonstrated significant increases across SCFAs, with concentrations being lowest in dry cows and highest in lactating cows (Figure 5A–G).

**Figure 6:**
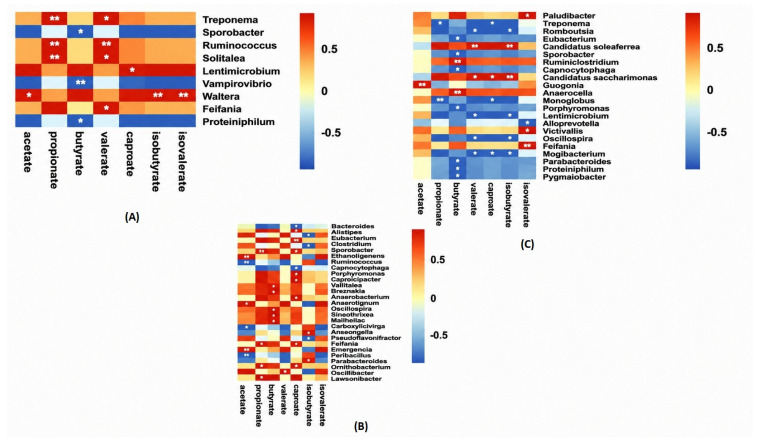
Correlation heat map analysis of relationships between seven types of SCFAs and genera in (**A**) Dry cows, (**B**) Lactating cows, (**C**) Pregnant cows. Trending red color means a positive correlation, and trending blue color means a negative correlation. *; significant correlation; **, extremely significant correlation.

Spearman’s correlation analysis was performed at the genus level to investigate the relationship between short-chain fatty acids (SCFAs) and gut microbiota in dry, lactating and pregnant cows, and the results are shown in the correlation heatmap (Figure 6A–C). In dry cows, *Guopingia* were positively linked to acetate; *Ruminiclostridium* and *Anaerocella* correlated positively with butyrate. Several genera (e.g., *Eubacterium*, *Sporobacter*, *Capnocytophaga*) were negatively associated with butyrate. *Treponema* and *Monoglobus* were negatively linked to propionate. In pregnant cows, *Waltera* were positively associated with acetate, isobutyrate, and isovalerate. *Ruminococcus*, *Treponema*, and *Solitalea* correlated positively with propionate and valerate. *Sporobacter*, *Vampirovibrio*, and *Proteiniphilum* were negatively correlated with butyrate. In lactating cows, *Ruminococcus*, *Oscillospira*, and *Emergencia* were positively associated with acetate. *Ethanoligenens*, *Feifania*, *Ornithobacterium*, and *Lawsinobacter* correlated positively with propionate. *Breznakia*, *Anaerobacterium*, *Spiroplasma*, *Kineothrix*, and *Maihella* were linked to butyrate. *Capnocytophaga*, *Carboxylicivirga*, and *Peribacillus* were negatively associated with acetate, while *Bacteroides* and *Porphyromonas* were negatively linked to caproate. *Eubacterium*, *Sporobacter*, and *Pseudoflavonifractor* were also negatively correlated with isobutyrate.

## 4. Discussion

Shannon and Simpson indices suggested that pregnant cows tended to have more diverse microbial communities, whereas dry and lactating cows showed lower diversity by these measures, although the differences were not statistically significant. Abundance-based Coverage Estimator (ACE) and Chao1 indices indicated a trend toward higher species richness in lactating cows and lower richness in pregnant cows, though these differences were also not statistically significant. Hormonal fluctuations during the estrous cycle and different reproductive stages likely influence this variation [32]. The number of observed species varied significantly across reproductive stages (*p* < 0.05), with lactating cows showing the highest richness and dry cows the lowest, aligning with previous findings of microbial differences between pregnant and non-pregnant cows [33]. Beta diversity analyses further demonstrated clear intergroup clustering of microbial communities, indicating that gut microbial communities of dry, lactating, and pregnant cows have statistically significant differences, but this effect is rather weak. This is consistent with previous studies [34], which showed that physiological status, diet, and sample type shape microbial structure.

At the phylum level, Bacillota and Bacteroidota, both dominant in ruminants, were abundant across all groups, confirming prior findings [23]. Bacillota were more prevalent in dry and lactating cows, although with no statistically significant difference, but lower in pregnant ones. This reflects shifts in energy metabolism and diet: Bacillota increase during pregnancy for energy storage and decline during lactation as the cow shifts to higher-starch diets to support milk production [35,36,37]. Bacteroidota were significantly more abundant in lactating cows and least abundant in dry cows. This phylum thrives on high-energy, fermentable diets typical of lactation, while lower abundance during the dry phase may result from forage-based diets and metabolic stress [38,39]. Bacteroidota play a role in fiber degradation and SCFA production, particularly propionate and butyrate, which support both energy metabolism and immune function [3]. The natural, shrub/grassland-based diet of Bunaji cows likely encourages the presence of Bacillota, Bacteroidota, and Fibrobacterota, which aid in breaking down coarse fiber into energy-rich SCFAs, promoting gut health and resilience in low-resource environments. The relative abundance of Pseudomonadota was significantly higher in dry cows and least abundant in pregnant cows. According to Zhang et al. [40], dry cows within a cattle herd exhibited a markedly higher Firmicutes presence, up to 95.8%, whereas Proteobacteria, including Pseudomonadota, showed a median level of only 0.29%. In contrast, pregnant cows displayed a significantly reduced abundance of Proteobacteria, reflecting the overall decline in Pseudomonadota during pregnancy. Lentisphaerota levels were significantly reduced in pregnant cows but reached their peak during lactation. This pattern aligns with earlier research by Marcos et al. [41], which reported greater Lentisphaerota abundance in late lactation compared to early lactation or the dry period. The variation in Lentisphaerota abundance between pregnancy and lactation may be attributed to shifts in the cow’s metabolic state during these stages, which affect the rumen environment and microbial communities [42].

The families Oscillospiraceae and Bacteroidaceae exhibited significantly higher abundance in lactating cows and the lowest abundance in pregnant cows. Oscillospiraceae (e.g., UCG-005) are linked to SCFA production and milk yield [43], but their levels drop under stress or during pregnancy. Bacteroidaceae increase significantly postpartum, particularly in early lactation [44]. Prevotellaceae are strongly associated with propionate production and milk volume and typically rise after calving [45]. Other key families such as Lachnospiraceae, Christensenellaceae and Clostridiaceae are involved in carbohydrate breakdown and SCFA synthesis [36,46,47]. These bacteria enhance fiber/starch digestibility and help maintain gut health. The relative abundance of Aristaeellaceae was significantly higher in pregnant cows and lowest in lactating cows.

The dominant genera included *Intestinimonas*, *Bacteroides*, *Acetivibrio*, and *Alistipes*, accounting for 11% of sequences in dry, 13% in lactating, and 9% in pregnant cows. This agrees with previous findings [44]. *Treponema* peaked in lactating cows, possibly due to dietary changes [48]. While *Alistipes* produce SCFAs, their abundance showed no clear pattern across stages [49]. *Bacteroides* appeared stable across stages in other mammals [50], though data on cows remain limited. Despite limited research on genus-level roles during pregnancy and lactation in cows, *Bacteroides*, *Intestinimonas*, and *Acetivibrio* are known to ferment carbohydrates and generate SCFAs [51,52,53]. The relative abundance of *Intestinimonas* and *Bacteroides* was significantly higher in lactating cows and lowest in pregnant cows. The higher abundance of *Intestinimonas* and *Bacteroides* in lactating cows may be due to increased energy demands and feed intake during lactation, which alter gut nutrient availability and favor the growth of carbohydrate- and protein-fermenting bacteria. Genera like *Bacteroides* are often noted among gut-associated taxa that fluctuate during early lactation or the transition period; for example, Zhu et al. [54] identified *Bacteroides* among gut-associated microbes present in milk, with their abundance shifting during the initial days of lactation.

LEfSe analysis identified key microbial genera associated with specific physiological states. *Intestinimonas* and *Bacteroides* were identified as biomarkers for lactating cows. *Bacteroides* species can metabolize bovine milk oligosaccharides, potentially affecting the calf’s gut microbiota [55,56]. *Intestinimonas* are commonly found in milk microbiota and may influence calf health [57,58]. *Aristaeella* was the dominant biomarker in pregnant cows, though its specific function remains unclear. However, it may contribute to pregnancy-related metabolic and immune processes [59,60].

The gut microbiota plays a central role in SCFA production. SCFA levels were highest in lactating cows and lowest in dry cows, indicating that pregnancy and its subsequent lactation may promote more active microbial metabolism [57]. Although only a few genera showed significant correlations with SCFA levels, possibly due to overlapping microbial functions, some associations were notable. In pregnant cows, *Ruminoclostridium* was positively associated with SCFAs, particularly acetate, propionate, and butyrate, corroborating the findings of Liu et al. [61], who reported that certain unclassified genera within the order Clostridiales were positively correlated (*p* < 0.05) with these SCFAs in cattle. In lactating cows, *Ruminococcus* and *Oscillospira* showed positive correlations with acetate production, consistent with previous findings [62,63]. As the current study is the first observational study of the fecal microbiota of this cattle tribe, these results could be validated in future studies by containing natural along with experimental diets, including several appropriate controls.

This study examined the gut microbial composition and its relationship with SCFAs in Bunaji cows across dry, lactating, and pregnant stages. To our knowledge, this is the first report on the gut microbiota of this cattle tribe. Despite that of all the alpha diversity indices, only the observed richness was statistically significant, pregnant cows exhibited slightly higher Shannon and Simpson indices. Beta diversity analyses revealed distinct clustering of microbial communities among groups. Bacillota and Bacteroidota were dominant phyla. Bacillota peaked in pregnant cows; Bacteroidota in lactating cows. At the family level, Oscillospiraceae and Bacteroidaceae were most abundant in lactating cows. *Intestinimonas* and Bacteroides were enriched in lactating cows and served as biomarkers. *Aristaeella* was dominant in pregnant cows. SCFA concentrations correlated positively with specific genera: *Ruminoclostridium* (lactating cows), *Ruminococcus* and *Oscillospira* (pregnant cows), and *Ruminococcus* and *Treponema* (dry cows). These findings highlight how gut microbiota adapt to meet energy and physiological demands across reproductive stages and offer valuable insights into microbial dynamics and metabolic interactions in cows during key physiological transitions.

## Figures and Tables

**Table 1:** Chemical composition of forages in natural grazing pasture (% dry matter basis).

Parameter	Composition (%)
Dry matter	90.2
Organic matter	89.9
Ash	10.1
Crude protein	15.4
Ether extract (lipids)	3.2
Total carbohydrates	71.2
Neutral detergent fiber	56.8
Acid detergent fiber	34.5
Non-fiber carbohydrates	14.6
Hemicellulose	22.2

**Table 2:** Alpha diversity indices of gut microbiota in dry, pregnant and lactating cows. SME: standard error of the mean.

Index	Dry	Lactating	Pregnant	SEM	*p*-Value
Observed	263.3	271.2	265.7	1.18	0.01
Chao1	274.0	276.3	270.1	5.09	0.24
ACE	270.1	274.2	270.3	6.92	0.16
Shannon	3.95	4.01	3.94	0.03	0.36
Simpson	0.96	0.96	0.96	0.002	0.44

**Table 3:** The relative abundance (%) of gut microbial communities at the phylum level in dry, lactating and pregnant groups.

Taxonomy	Dry	Lactating	Pregnant	SEM	*p*-Value
Bacillota	59.28	54.94	59.37	1.270	0.281
Bacteroidota	25.46 ^b^	32.71 ^a^	27.99 ^ab^	1.132	0.018
Verrucomicrobiota	4.71	4.13	5.03	0.993	0.939
Spirochaetota	3.46	3.97	2.33	0.318	0.090
Pseudomonadota	2.50 ^a^	0.73 ^b^	1.92 ^ab^	0.297	0.036
*Candidatus* Saccharibacteria	1.24	0.21	0.93	0.220	0.148
Mycoplasmatota	0.71	0.62	0.52	0.059	0.452
Lentisphaerota	0.55 ^ab^	0.82 ^a^	0.18 ^b^	0.106	0.034
Thermodesulfobacteriota	0.47	0.67	0.35	0.065	0.138
Cyanobacteriota	0.34	0.36	0.40	0.048	0.866

SEM, standard error of the mean. Means in a row with different small-letter superscripts differ significantly (*p* < 0.05); same-letter superscripts indicate no differences (*p* > 0.05).

**Table 4:** The relative abundance (%) of gut microbial communities at the family level in dry, lactating and pregnant groups.

Taxonomy	Dry	Lactating	Pregnant	SEM	*p*-Value
Oscillospiraceae	28.02 ^a^	30.34 ^a^	21.76 ^b^	1.316	0.012
Bacteroidaceae	6.45 ^b^	10.61 ^a^	5.49 ^b^	0.678	<0.001
Sphingobacteriaceae	6.97	5.47	5.66	0.595	0.562
Rikenellaceae	4.81	5.37	8.70	1.412	0.506
Lachnospiraceae	5.30	4.57	4.33	0.188	0.081
Akkermansiaceae	4.67	3.55	4.94	1.034	0.860
Paludibacteraceae	3.46	4.48	3.49	0.244	0.154
Caryophanaceae	2.68	1.30	5.31	0.773	0.092
Treponemataceae	3.58	3.64	2.28	0.308	0.120
Aristaeellaceae	2.95 ^a^	1.61 ^b^	3.16 ^a^	0.280	0.039

SEM, standard error of the mean. Means in a row with different small-letter superscripts differ significantly (*p* < 0.05); same-letter superscripts indicate no differences (*p* > 0.05).

**Table 5:** The relative abundance (%) of gut microbial communities at the genus level in dry, lactating and pregnant groups.

Taxonomy	Dry	Lactating	Pregnant	SEM	*p*-Value
*Intestinimonas*	7.39 ^b^	9.85 ^a^	4.87 ^c^	0.631	<0.001
*Bacteroides*	6.03 ^b^	10.02 ^a^	5.43 ^b^	0.645	0.001
*Acetivibrio*	6.27	6.33	5.49	0.215	0.209
*Lysinibacillus*	6.10	3.03	7.75	1.136	0.237
*Akkermansia*	4.47	3.41	4.97	0.994	0.824
*Parapedobacter*	4.82	3.64	4.37	0.374	0.824
*Alistipes*	3.87	4.49	3.27	0.246	0.127
*Paludibacter*	3.28	4.35	3.52	0.233	0.144
*Solibacillus*	2.49	1.24	5.58	0.778	0.054
*Treponema*	3.40	3.54	2.29	0.294	0.169

SEM, standard error of the mean. Means in a row with different small-letter superscripts differ significantly (*p* < 0.05); same-letter superscripts indicate no differences (*p* > 0.05).

## Data Availability

Raw sequence reads have been deposited in the Short Read Archive (https://www.ncbi.nlm.nih.gov/sra; BioProject access number PRJNA1439808).
